# The Prognostic Values of Leukocyte Rho Kinase Activity in Acute Ischemic Stroke

**DOI:** 10.1155/2014/214587

**Published:** 2014-02-27

**Authors:** Cheng-I. Cheng, Yu-Chun Lin, Tzu-Hsien Tsai, Hung-Sheng Lin, Chia-Wei Liou, Wen-Neng Chang, Cheng-Hsien Lu, Chun-Man Yuen, Hon-Kan Yip

**Affiliations:** ^1^Division of Cardiology, Department of Internal Medicine, Kaohsiung Chang Gung Memorial Hospital, No. 123, Ta-Pei Road, Niao-Song, Kaohsiung City 83301, Taiwan; ^2^Chang Gung University College of Medicine, Kaohsiung City 83301, Taiwan; ^3^Department of Neurology, Kaohsiung Chang Gung Memorial Hospital, Kaohsiung City 83301, Taiwan; ^4^Division of Neurosurgery, Department of Surgery, Kaohsiung Chang Gung Memorial Hospital, Kaohsiung City 83301, Taiwan

## Abstract

*Objective*. It has been reported that leukocyte ROCK activity is elevated in patients after ischemic stroke, but it is unclear whether leukocyte ROCK activity is associated with clinical outcomes following acute stroke events. The objective of this study is to investigate if leukocyte ROCK activity can predict the outcomes in patients with acute ischemic stroke. *Materials and Methods*. We enrolled 110 patients of acute ischemic stroke and measured the leukocyte ROCK activity and plasma level of inflammatory cytokines to correlate the clinical outcomes of these patients. *Results*. The leukocyte ROCK activity at 48 hours after admission in acute ischemic stroke patients was higher as compared to a risk-matched population. The leukocyte ROCK activity significantly correlated with National Institute of Health Stroke Scale (NIHSS) difference between admission and 90 days after stroke event. Kaplan-Meier survival estimates showed lower stroke-free survival during follow-up period in patients with high leukocyte ROCK activity or plasma hsCRP level. Leukocyte ROCK activity independently predicted the recurrent stroke in patients with atherosclerotic stroke. *Conclusions*. This study shows elevated leukocyte ROCK activity in patients with ischemic stroke as compared to risk-matched subjects and is an independent predictor for recurrent stroke.

## 1. Introduction

The pathogenesis of ischemic stroke includes embolism, atherosclerosis, thrombus formation, infarction-related necrosis, and reperfusion injury after recanalization [1]. In addition to conventional cardiovascular disease (CVD) risk factors, a variety of inflammatory cytokines or biomarkers are now considered to contribute to the development of ischemic stroke [2]. Indeed, it has been proven that atherosclerotic lesions contain monocyte-derived macrophages and T lymphocytes [3] and leukocytosis is associated with plaque thickness in the carotid artery [4]. Moreover, leukocytes may jeopardize the integrity of the endothelium which is important for the function of the blood brain barrier [5]. There is accumulating evidence that leukocyte-mediated vascular inflammation is implicated in the pathogenesis of ischemic stroke.

Rho-associated kinase (ROCK) is the downstream target of small GTP-binding protein RhoA GTPase and is upregulated by inflammatory stimuli, such as angiotensin II and interleukin-1**β** [6]. ROCK plays an important role in a variety of cellular functions, including cytoskeleton [7], migration [8], and contraction [9], and inhibition of ROCK attenuates recruitment and adhesion of leukocytes [10]. In particular, ROCK may attenuate the expression of endothelial nitric oxide synthase in endothelial cells, and inhibition of ROCK may suppress the proliferation of vascular smooth muscle cells and foam cell formation derived from macrophage [11]. Additionally, Rho/ROCK activation by C-reactive protein has been reported to enhance plasmogen activator inhibitor-1, which may result in atherothrombogenesis [12]. More and more studies suggest that Rho/ROCK activation is a major pathway in vascular inflammation.

Unlike the well-established role of high sensitive C-reactive protein (hsCRP) in the development of cardiovascular disease [13], the prognostic value of leukocyte ROCK activity in cardiovascular disease is less investigated. Previous studies have shown that human leukocyte ROCK activity is associated with metabolic syndrome [14] and endothelial dysfunction [15] and that statins can inhibit human leukocyte ROCK activity in patients with established atherosclerosis [16]. Interestingly, it has been reported that leukocyte ROCK activity is elevated in patients within 48 hours after ischemic stroke [17]. However, it is not known whether leukocyte ROCK activity is associated with outcomes after acute ischemic stroke. In this study, we prospectively enrolled patients of acute ischemic stroke and measured their leukocyte ROCK activity and plasma hsCRP level to correlate the clinical outcomes of these patients.

## 2. Materials and Methods

### 2.1. Study Subjects

After receiving approval (96-1381A; 99-3438C) from the Institutional Review Board of Kaohsiung Chang Gung Memorial Hospital, we prospectively enrolled 110 patients with acute ischemic stroke that had been admitted to the Department of Neurology from November 2008 to October 2011, and also a risk-matched control group without stroke history was derived from the Health Examination Center at our hospital. All study subjects provided informed consent. Clinical information was collected based on the review of medical records upon admission and during follow-up.

### 2.2. Preparation of Plasma

Venous whole blood samples were collected from each subject into a heparinized tube (BD) at 48 hours after admission after overnight fasting. Cells were removed from plasma by centrifugation for 10 minutes at 2,000 g. The resulting supernatant (designated plasma) was immediately transferred into a clean polypropylene tube following centrifugation. Then the plasma samples were apportioned into 0.5 mL aliquots and stored at −80°C until biochemical assay was conducted.

### 2.3. Leukocyte Isolation

According to manufacturer's instruction, a 9 mL heparin-containing blood sample was carefully layered on a 6 mL Ficoll-Paque PLUS (GE Healthcare Bioscience Corp., Pittsburgh, PA) in a 15 mL centrifuge tube and was centrifuged at 400 g for 40 minutes at 20°C. After centrifuge, the upper layer was drawn off and the leukocyte layer at the interface was collected carefully and resuspended in another centrifuge tube containing 10 mL Hank's balanced salt solution (Invitrogen/GIBCO, Carlsbad, CA). Then the leukocyte samples were centrifuged at 100 g for 10 minutes at 20°C, and the supernatant was discarded. This wash step was repeated once to remove the residual Ficoll-Paque PLUS. The lymphocytes were then diluted with Hank's balanced salt solution to achieve 5 × 10^6^ cells/mL and apportioned into 400 *μ*L cell aliquots in 4 sterile 1.5 mL microcentrifuge tubes. We added 100 *μ*L of fixative solution containing 50% trichloroacetic acid (Sigma, St. Louis, MO), 50 mM dithiothreitol (Sigma), and protease inhibitors (Calbiochem, UK) to each tube. After vortexing and centrifuging the samples at 4°C for 5 min at 11,000 g, the supernatant was removed. The samples were centrifuged again at 4°C for 1 min at 11,000 g to remove the residual supernatant. The leukocyte pellets were stored at −80°C till use.

### 2.4. Cytokine Analysis

Plasma levels of hsCRP, tumor necrotic factor alpha (TNF*α*), and interleukin-6 (IL-6) were measured by commercially available enzyme-linked immunosorbent assay kits (R&D, Minneapolis, MN) according to manufacturer's instructions. All the experiments were repeated in duplicate.

### 2.5. Assay for Leukocyte ROCK Activity

The assay for leukocyte ROCK activity was performed based on previous study [18]. The frozen cell pellets were resuspended in 20 *μ*L of 1 M Tris and then mixed with 200 *μ*L of extraction buffer (8 M urea, 2% sodium dodecyl sulfate, 5% sucrose, 5% **β**-mercaptoethanol, 0.02% bromophenol blue). After boiled on heat block at 95°C, the samples were cooled down on ice and centrifuged briefly at room temperature for 30 seconds at 11,000 g. Then, 20 *μ*L sample of each study subject was loaded to 8% sodium dodecyl sulfate polyacrylamide gel electrophoresis and transferred onto polyvinylidene fluoride membrane (Millipore, Bedford, MA). The same batch of cell lysates derived from RAW 264.7 cell line were processed following the protocol mentioned above as an external control to standardize the results of Western blot analyses from different membranes. After transfer, the polyvinylidene fluoride membranes were incubated in 3% bovine serum albumin at room temperature and then overnight at 4°C with rabbit primary polyclonal antibodies against phospho-specific Thr^853^-myosin binding subunit (MBS) (Abcam Cambridge, MA) (1 : 2000), or MBS (Covance, Berkley, CA) (1 : 1000). The membranes were washed and incubated for 1.5 hours with horseradish peroxidase-labeled donkey anti-rabbit antibodies (GE healthcare). Immunoreactive bands were visualized by enhanced chemiluminescent detection reagent (Perkin Elmer, Boston, MA). Leukocyte Rho kinase activity was expressed as the ratio of phosphorylation levels of MBS (pMBS) in each sample per pMBS in each positive control divided by total MBS (tMBS) in each sample per tMBS in each external control.

### 2.6. Evaluation of Pharmaceutical Agents on *In Vitro* ROCK Activity of Leukocyte

In some experiments, leukocytes derived from nonstroke subjects were isolated and kept in M199 medium before ROCK assay. Based on previous studies [19–22], 1 × 10^7^ leukocytes were subjected to the treatment of either 0.01% DMSO for 30 minutes, 10 *μ*M valsartan for 30 minutes, 10 *μ*M pioglitazone for 60 minutes, 10 *μ*M atorvastatin for 30 minutes, or 10 *μ*M amlodipine for 30 minutes, respectively, followed by 10 *μ*M lysophosphatidic acid (LPA) [23] for 10 min. *In vitro* ROCK activities of these treated leukocytes were then examined as described above. All of these agents were purchased from Sigma Aldrich.

### 2.7. Statistical Analysis

Continuous variables were expressed as mean ± SD (normal distribution) or median with quartile range (not normal distribution), and categorical variables were expressed as frequencies. The frequencies between the groups were compared with chi-square analysis. An independent-samples *t* test was used for comparison of parametric variables, whereas Mann-Whitney *U* test was used to for analysis of nonparametric variables. Because of the great difference in the baseline characteristics between the control group and the stroke group, we calculated propensity scores predicting conditional probabilities to avoid selection bias. The covariates that were adjusted for risk matching included sex, age, diabetes, hypertension, dyslipidemia, body mass index, and smoking, and only 33 pairs were matched from 110 patients in the stroke group and 118 patients in the control group. Spearman correlation test was performed to analyze the correlation between leukocyte ROCK activities, hsCRP, IL-6, and TNF*α*. Receiver-operating characteristic analysis was conducted to determine the best cut-off value of leukocyte ROCK activity for prediction of recurrent stroke during follow-up. Distributions of recurrent stroke events during follow-up were estimated using the Kaplan-Meier method, and intergroup differences were compared using the log rank test. Cox proportional hazard model was performed for multivariate analysis of predictors for recurrent stroke during follow-up. The covariates controlled for confounding effect included age, sex, dyslipidemia, diabetes, hypertension, NIHSS on admission, WBC count, leukocyte ROCK activity, hsCRP, IL-6, and TNF*α*. A two-tailed *P* value <0.05 was considered statistically significant with a 95% confidence interval. All statistical analyses were performed using SPSS 13.0.

## 3. Results

### 3.1. Higher Leukocyte ROCK Activity in Acute Ischemic Stroke Patients

**Figure 1:**
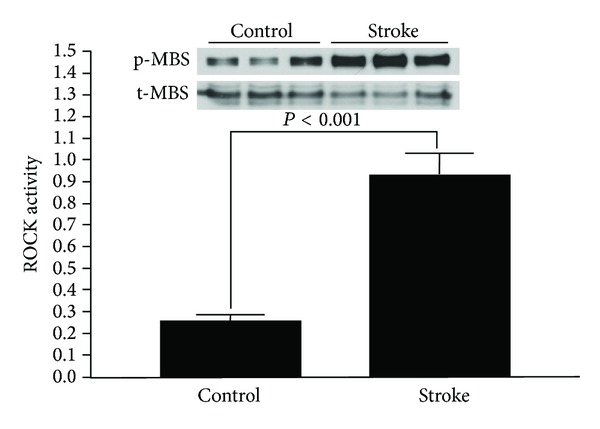
Leukocyte ROCK activity in acute ischemic stroke patients and risk-matched subjects. The bar chart displays the relative leukocyte ROCK activity in acute ischemic stroke patients (*n* = 33) and risk-matched subjects (*n* = 33) as indicated. The representative blot in the panel demonstrates the Western blot of p-MBS and t-MBS of three patients in each group.

To determine if leukocyte ROCK activity is elevated in acute ischemic stroke patients, we compared the leukocyte ROCK activity in patients of ischemic stroke selected by propensity-score matching (*n* = 33) with those in CVD risk-matched group without apparent cardiovascular morbidity (*n* = 33).  Table 1 lists the demographic data of both groups. No significant difference was noted in the major cardiovascular risk factors between the two groups, and the average numbers of risk factors were similar in both groups.  Figure 1 shows that the leukocyte ROCK activity of ischemic stroke group was significantly higher than that in the risk-matched control group (median ± quartile: 0.935 ± 0.602 versus 0.258 ± 0.175, *P* < 0.001). These results indicate that leukocyte ROCK activity was higher in patients of acute ischemic stroke than those with similar CVD risk factors but with no stroke.

### 3.2. Demographics of Ischemic Stroke Patients

To analyze the relationship between leukocyte ROCK activity and outcomes following the acute stroke episode, we further analyzed the data of 110 patients with acute ischemic stroke, and Table 2 shows the demographics of this patient cohort. The mean NIHSS at admission was 6.4 ± 6.8 (median ± quartile: 4.0 ± 2.8).

### 3.3. Determination of Cut-Off Values for ROCK Activity and hsCRP

To investigate whether leukocyte ROCK activity upon admission can predict recurrent stroke after an acute ischemic stroke event, we conducted a receiver-operating characteristic (ROC) plot to determine the cut-off values of leukocyte ROCK activity and plasma hsCRP level for recurrent stroke. The area under curve of hsCRP level and ROCK activity was 0.691 (*P* = 0.060) (Supplemental Figure 1(a)) and 0.704 (*P* = 0.021) please see the Supplementary Materials available at http://dx.doi.org/10.1155/2014/214587 (Supplemental Figure 1(b)), respectively. We then used a plasma hsCRP level of 2.80 (sensitivity: 0.667; specificity: 0.686) and leukocyte ROCK activity of 0.56 (sensitivity: 0.704; specificity: 0.750) as the cut-off values for predicting recurrent stroke and stratified our patients into either low or high leukocyte ROCK activity groups and low or high plasma hsCRP groups based on these values.

### 3.4. Correlation of Leukocyte ROCK Activity and Stroke Outcome Parameters

**Figure 2:**
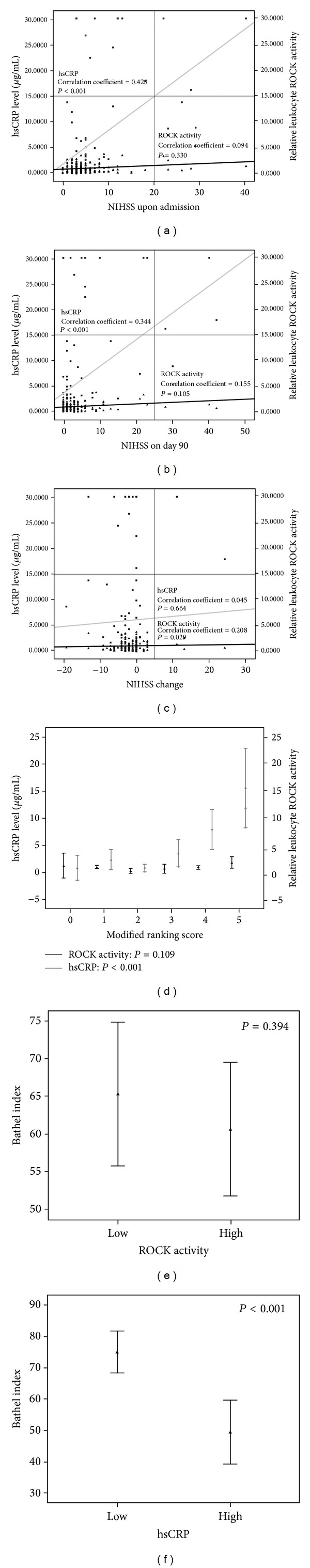
Correlation of hsCRP level and ROCK activity with neurological function scales in patients with stroke. (a) Correlation with NIHSS upon admission. (b) Correlation with NIHSS on 90th day. (c) Correlation with NIHSS changes between admission and 90th day. (d) Plasma hsCRP level and leukocyte ROCK activity grouped by modified Rankin score. (e) Barthel index in patients with high and low plasma hsCRP level. (f) Barthel index in patients with high and low leukocyte ROCK activity. ▲: leukocyte ROCK activity; ■: plasma hsCRP level; black solid line: regression curve of leukocyte ROCK activity; gray solid line: regression curve of plasma hsCRP level.

Previous studies have shown baseline NIHSS, 90-day NIHSS, the difference between baseline and 90th day NIHSS, and 90-day modified Rankin score correlates with early reperfusion, subsequent improvement or deterioration in neurological symptom, and mortality [24, 25]. To investigate if the leukocyte ROCK activity and hsCRP in ischemic stroke patients were associated with NIHSS, modified Rankin score, or Barthel index, we conducted the Spearman correlation test, and the results are displayed in Figure 2. The leukocyte ROCK activity did not associate with NIHSS upon admission (Figure 2(a)) and NIHSS in 90 days (Figure 2(b)). The plasma hsCRP level in acute ischemic stroke patients was significantly correlated with NIHSS upon admission (*P* < 0.001, correlation coefficient = 0.428) and on 90th day (*P* < 0.001, correlation coefficient = 0.344). The leukocyte ROCK activity significantly but not hsCRP correlated with NIHSS difference between admission and 90th day after stroke event (*P* = 0.029, correlation coefficient = 0.208) (Figure 2(c)). Furthermore, higher modified Rankin score on day 90 is significantly associated with higher plasma hsCRP level (*P* < 0.001) but not the leukocyte ROCK activity (*P* = 0.109) (Figure 2(e)). We further used the median values of ROCK activity (median ± standard error of mean = 0.772 ± 0.893) and plasma hsCRP (median ± standard error of mean = 1.739 ± 8.420) as cut-off points to stratify patient into high or low ROCK activity and plasma hsCRP groups, respectively. Barthel index of patients was significantly different between high and low hsCRP groups (*P* < 0.001) (Figure 2(f)). However, no difference in Barthel index was noted between high and low ROCK groups (*P* = 0.394) (Figure 2(e)). Our results show that leukocyte ROCK activity upon admission significantly correlates with NIHSS change while hsCRP is associated with neurological deficits at baseline and on 90th day after acute ischemic stroke.

### 3.5. Correlation of Leukocyte ROCK Activity and Inflammatory Cytokine

Because white blood cell (WBC) count, hsCRP, TNF*α*, and IL-6 are reported to be independent parameters predicting stroke outcomes [26–28], we are interested in whether leukocyte ROCK activity correlates with these inflammatory cytokines in acute ischemic stroke patients. We conducted the Spearman correlation test to examine the correlation between leukocyte ROCK activity, hsCRP, TNF*α*, and IL-6, and the results are shown in Table 3. Leukocyte ROCK activity did not correlate with WBC count or serum level of hsCRP, IL-6, or TNF*α*. However, IL-6 significantly correlated with TNF*α* (correlation efficiency = 0.242; *P* = 0.016) and hsCRP (correlation efficiency = 0.565; *P* < 0.001). These results suggest that the elevation of leukocyte ROCK activity in acute ischemic stroke patients is independent of WBC count, hsCRP, IL-6, or TNF*α*.

### 3.6. Both Leukocyte ROCK Activity and Plasma hsCRP Level Predict Recurrent Stroke

**Figure 3:**
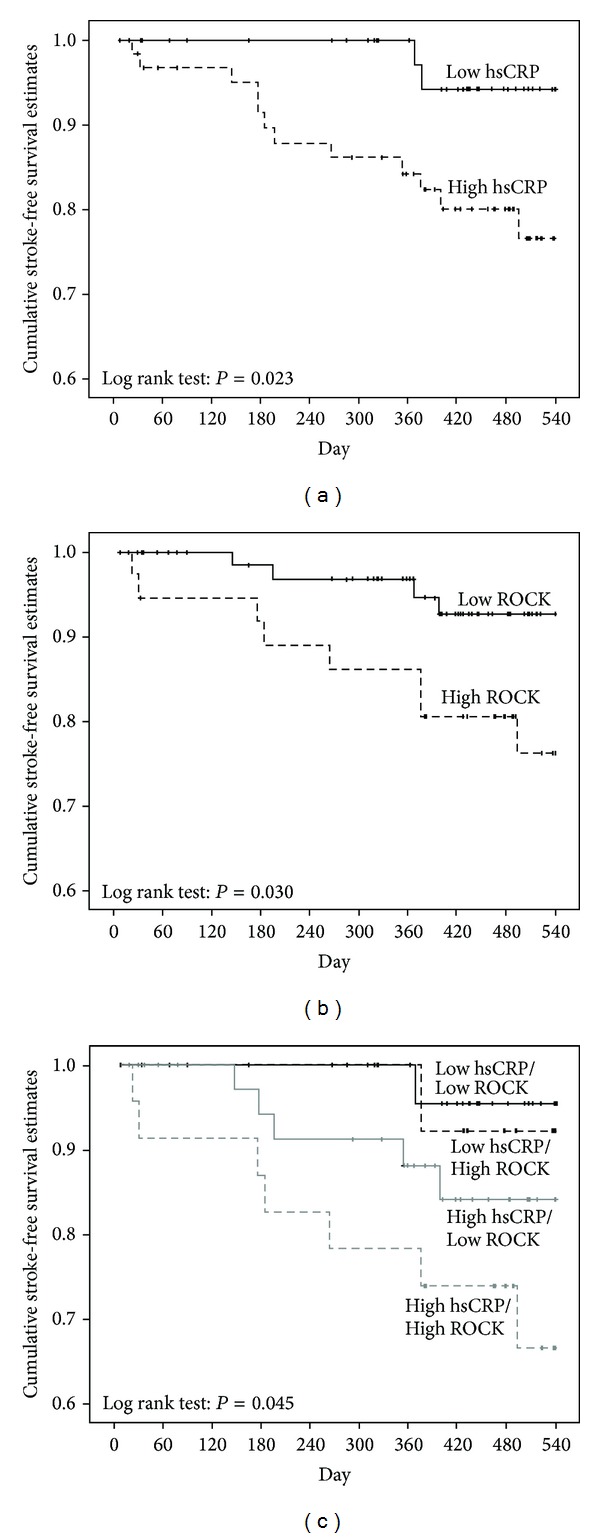
Kaplan-Meier survival estimates of stroke-free survival during follow-up. (a) Survival estimate of stroke-free survival in patients of high and low hsCRP groups. Solid line: low hsCRP group; dashed line: high hsCRP group. (b) Survival estimate of stroke-free survival in patients of high and low leukocyte ROCK activity groups. Solid line: low leukocyte ROCK activity group; dashed line: high leukocyte ROCK activity group. (c) Survival estimate of stroke-free survival by grouped by plasma hsCRP and leukocyte ROCK activity. Black solid line: low hsCRP and low ROCK activity; black dashed line: low hsCRP and high ROCK activity; gray solid line: high hsCRP and low ROCK activity; gray dashed line: high hsCRP and high ROCK activity.

To clarify if plasma hsCRP level and leukocyte ROCK activity upon admission can predict recurrent stroke after an acute ischemic stroke event, we conducted a survival analysis. Kaplan-Meier survival estimates of the entire ischemic stroke patient cohort showed a significantly higher rate of recurrent stroke during follow-up in the high hsCRP group as compared to that in the low hsCRP group (*P* = 0.023) (Figure 3(a)), and the patients in the high leukocyte ROCK activity group also had lower stroke-free survival (*P* = 0.030) (Figure 3(b)). When grouped by hsCRP and ROCK activity, the patients with high hsCRP level and high ROCK activity had the lowest stroke-free survival, followed by high hsCRP level and low ROCK activity, low hsCRP level and high ROCK activity, and low high hsCRP level and low ROCK activity (*P* = 0.045) (Figure 3(c)). Interestingly, Cox regression analysis of the entire ischemic stroke cohort (*n* = 110) revealed that hsCRP was the only significant predictor for recurrent stroke (hazard ratio: 1.214; 95% confidence interval: 1.068–1.381; *P* = 0.003) while leukocyte ROCK activity was not an independent predictor for recurrent stroke (hazard ratio: 1.828; 95% confidence interval: 0.536–6.289; *P* = 0.338) after adjustment of other potential confounding factors. Nevertheless, Cox regression analysis of the atherosclerotic stroke patients, who were classified as microangiopathy or macroangiopathy of Trial of Org 10172 in Acute Stroke Treatment etiology (=89), showed that both hsCRP (hazard ratio: 1.351; 95% confidence interval: 1.056–1.728; *P* = 0.017) and leukocyte ROCK activity (hazard ratio: 5.207; 95% confidence interval: 1.200–22.584; *P* = 0.028) were significant predictors for recurrent stroke (Table 4). These results indicate that plasma hsCRP level upon admission for acute ischemic stroke is associated with recurrent stroke in both atherosclerotic and nonatherosclerotic stroke patients during long-term follow-up while leukocyte ROCK activity correlates with the recurrent stroke only in patients who had atherosclerotic stroke.

### 3.7. The Effects of Cardiovascular Medications on *In Vitro* Leukocyte ROCK Activity

**Figure 4:**
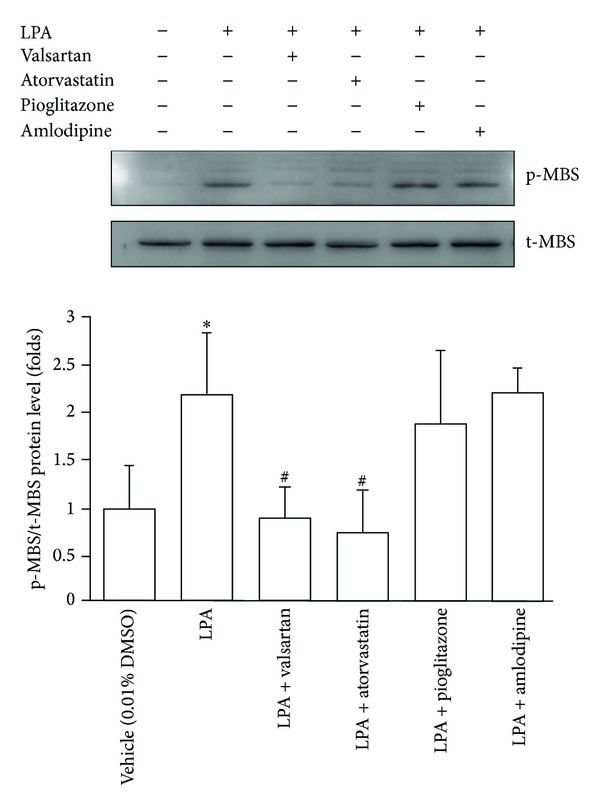
Effect of cardiovascular medications on *in vitro* ROCK activity in human leukocytes. Representative immunoblotting and density plot of Rho kinase activity in human leukocytes (expressed as p-MBS/t-MBS ratio) under the treatment of pharmaceutical agents as indicated. LPA was used as positive control for Rho kinase activation. *: *P* < 0.05 versus vehicle. ^#^: *P* < 0.05 versus LPA. LPA: lysophosphatidic acid.

We are interested in whether some of cardiovascular medications may change leukocyte ROCK activity in these patients. LPA is a positive regulator of ROCK. The plasma level of LPA is elevated in patients with ischemic cerebral vascular diseases and acute myocardial infarction [29–31], and LPA is found to accumulate in human atherosclerotic lesion [32]. Therefore, we treated human leukocytes derived from healthy subjects with atorvastatin, valsartan, pioglitazone, or amlodipine to evaluate their direct effects on *in vitro* LPA-accentuated ROCK activities. Interestingly, both atorvastatin and valsartan can significantly attenuate *in vitro* LPA-induced ROCK activation in human leukocytes (Figure 4). Neither amlodipine nor pioglitazone can change *in vitro* ROCK activity in leukocytes. These results suggest that some commonly used cardiovascular medications may potentially change leukocyte activity.

## 4. Discussion

Our study showed that leukocyte ROCK activity is not only correlated with NIHSS change in 90 days after an acute ischemic stroke event but also associated with lower stroke-free survival, while plasma hsCRP level is related to the severity of neurological dysfunction at acute stage and is an independent predictor for recurrent stroke. To the best of our knowledge, this is first time that leukocyte ROCK activity has been proven to correlate with outcomes following acute ischemic stroke.

### 4.1. Plasma hsCRP Level and Leukocyte ROCK Activity in Cardiovascular Diseases

Many biomarkers have been investigated to be the predictor of outcomes after ischemic stroke [33]. Plasma hsCRP is one of those to be of great prognostic value in a variety of cardiovascular diseases but the role of leukocyte ROCK activity in cardiovascular diseases is less investigated. Recent studies have shown that higher leukocyte ROCK activities are observed in smokers [15], metabolic syndrome [14], and patients with endothelial dysfunction [34]. Patients with established cardiovascular disease [35] or chronic heart failure [36] also have higher leukocyte ROCK activity than a normal population. Similar to our study, one previous study indicated that higher leukocyte activities in acute ischemic stroke as compared to CVD risk-matched population and the elevated leukocyte ROCK activity may persist for 48 hours after the onset of stroke [17]. Together with the results of our study, these lines of evidence suggest that leukocyte ROCK activity may be elevated along with the progression of atherosclerosis.

### 4.2. Local ROCK Activation in Cerebral Infarction

In addition to elevated human leukocyte ROCK activity in acute ischemic stroke patients, one animal study using rat cerebral infarction model also demonstrated that cerebral ROCK activity was elevated in ipsilateral cerebral hemisphere than that in contralateral cerebral hemisphere [37]. Indeed, several studies have proven that the administration of ROCK inhibitor, fasudil, or hydroxyfasudil significantly attenuated the infarction size, induced neurogenesis, and improved the cognitive function in murine cerebral ischemia or an infarction model [38]. The mechanisms that have been addressed in ROCK-mediated brain ischemic injury include the generation of reactive oxygen species [39], the inhibition of endothelial nitric oxide synthase activity in cerebral vascular endothelial cells [40], and the enhancement of migration of neural stem cells [8]. Further studies are warranted to investigate if ROCK inhibitor can improve neurological function following acute ischemic stroke.

### 4.3. Potential Mechanisms Linking Leukocyte ROCK Activity and Ischemic Stroke

While leukocyte is considered to be involved in the pathogenesis of atherosclerosis and cerebral infarction, ROCK plays an important role in leukocyte functions, including cytokine formation, recruitment, and migration [41, 42]. One previous study demonstrated that the isoform ROCK1 is crucial for macrophage-mediated atheroma formation [11], indicating that the activation of ROCK is important in the inflammation process of atherosclerosis. Furthermore, the major risks of ischemic stroke are within the scope of metabolic syndrome, and inflammatory cytokines including hsCRP, TNF*α*, and IL-6 have been proven to be significantly associated with the occurrence and outcomes of ischemic stroke [27]. It has been shown that the leukocyte ROCK activity in patients of metabolic syndrome is proportional to the number of metabolic syndrome components and associated with higher plasma level of hsCRP [14]. These lines of evidence suggest that ROCK in leukocyte may potentially contribute to the vascular inflammation and subsequent ischemic stroke events. Additionally, the changes of NIHSS between the baseline and 90th day have been adopted as an endpoint in several clinical trials of treatment for acute ischemic stroke [43, 44]. Indeed, one human study has shown that infarct growth, defined as volume difference between baseline diffusion-weighted imaging and 90th day fluid-attenuated inversion recovery image lesions on brain magnetic resonance image, correlated with NIHSS score change from baseline to 90th day [45]. Our study demonstrated that leukocyte ROCK activity is associated with NIHSS change between the baseline and 90th day after ischemic stroke, suggesting that the leukocyte ROCK activity upon acute ischemic stroke may be inversely related to recovery of the neurological deficit caused by cerebral infarction and may play an important role in the remodeling process of the cerebral infarct area.

### 4.4. Leukocyte ROCK Activity and Stroke Etiology

Our study fails to demonstrate the correlation between leukocyte ROCK activity and various inflammatory cytokines. Additionally, our analysis showed that hsCRP is the only independent predictor for recurrent stroke in the entire ischemic stroke patient cohort whereas leukocyte ROCK activity independently correlates with the recurrent stroke only in patents with atherosclerotic stroke. Although ROCK has been proved to be involved in platelet activation [46], ROCK was only reported to be involved in the atherosclerosis rather than cerebral embolism and hemorrhage. Further studies should be conducted to adjust for these potential confounders and to elucidate the relationship between inflammatory cytokines and leukocyte ROCK activity in patients with acute ischemic stroke.

### 4.5. Cardiovascular Medications and Stroke Outcomes

Early thrombolytic therapy is one of the most important pharmaceutical interventions to improve outcomes after acute ischemic stroke [47]. Additionally, mediations for treatment of hypertension and glycemic control have been proved to reduce inflammatory cytokine, inhibit platelet activity and procoagulant status, and improve long-term cardiovascular outcome after ischemic stroke [48, 49]. Hypertension is an important and popular risk favor for the stroke, and reduction of blood pressure is proportional to the risk reduction in secondary prevention of stroke. However, hypertensive patients receiving beta blockers have a higher incidence of stroke when compared to other antihypertensive agents in the meta-analysis [50]. Furthermore, compared to angiotensin-converting enzyme inhibitors (ACEIs), calcium channel blockers (CCB) prevent more stroke events but fewer myocardial infarction events per reduction of one mmHg in systolic blood pressure [51]. On the other hand, a prospective randomized control trial comparing amlodipine and valsartan in patients with high cardiovascular risk shows comparative results in primary composite endpoints and occurrence of stroke although amlodipine prevents more stroke events in the first 3 months of the study due to more rapid reduction of blood pressure compared to valsartan [52]. Therefore, treatment in acute stage and choice of antihypertensive agents might potentially affect the outcomes after ischemic stroke.

### 4.6. Cardiovascular Medications and Leukocyte ROCK Activity

Previous studies have shown that *in vivo* human leukocyte ROCK activity could be affected by lipid-lowering agents and antihypertensive agents, including statins [34], CCBs [35], and mineralocorticoid receptor blocker [53]. Nevertheless, whether these medications exert their effects directly on leukocyte or through other mechanisms is unclear. One cross-sectional study has showed that calcium channel blocker but not RAS blockade can reduce *in vivo* leukocyte ROCK activity in hypertensive patients [35]. However, our study shows that, instead of amlodipine, atorvastatin and valsartan attenuate *in vitro* LPA-induced ROCK activation in human leukocytes derived from health subjects. It has been shown that benidipine (CCB) attenuates vasodilation induced by Y-27632 (ROCK inhibitor) in spontaneous hypertensive rats [54], whereas another study demonstrates that losartan counteracts the hyperreactivity to angiotensin II and ROCK1 over-activation in aortas isolated from streptozotocin-injected diabetic rats [55]. Furthermore, both losartan and amlodipine can attenuate the production of TGF-*β* and TNF-*α* in human mononuclear cell culture [56]. The discrepancy between the results of our *in vitro* study and those of T. Hata et al. may be due to (1) the heterogeneity in dose and type of antihypertensive medications prescribed to the patients, (2) human leukocytes derived from hypertensive patients of different period of hypertension and treatment rather than those from health subjects under the same *in vitro* stimulation of LPA, and (3) *in vivo* cross-sectional comparison of leukocyte ROCK activity between different treatment groups instead of *in vitro* direct verification of the effects of these pharmaceutical agents on the leukocyte derived from the same study subjects. Together with the results of the current and previous studies, the mechanism underlying CCB attenuates *in vivo* leukocyte ROCK activity in hypertensive patients might not directly go through the interaction of calcium ion channel and upstream ROCK pathway in leukocytes. Further studies are warranted to investigate this interesting phenomenon.

### 4.7. Clinical Implications

Our study showed that leukocyte ROCK activity is associated with NIHSS change in 90 days and recurrent stroke after acute ischemic stroke, especially those with atherosclerotic stroke. Given that the measurement of cerebral ROCK activity is not feasible in clinical practice, leukocyte ROCK activity may be used as a biomarker to predict short-term improvement of neurological function. Moreover, plasma hsCRP level predicts neurological dysfunction at the acute stage and recurrent stroke during follow-up. Therefore, for those patients with high leukocyte ROCK activity and plasma hsCRP, more aggressive intervention is necessary to achieve optimal targets of blood pressure, glucose, and lipid in order to restore neurological function and prevent recurrent stroke.

### 4.8. Study Limitations

The major limitations of this study were small sample size and that all the subjects enrolled in this study were from a single hospital and serial measurement of the leukocyte ROCK activity was not performed. In addition, the underlying mechanism with regard to elevated leukocyte ROCK activity in patients of ischemic stroke was not investigated. Finally, some pharmaceutical interventions regarding CVD risk factors and stroke may confound our analysis of the relationship between leukocyte ROCK activity, inflammatory cytokines, and outcomes but were not controlled during follow-up. Nevertheless, our findings show that, in addition to plasma hsCRP level, leukocyte ROCK activity is an important biomarker of acute ischemic stroke and may help to predict outcomes following this event.

## 5. Conclusion

Our study showed higher leukocyte ROCK activity in acute ischemic stroke patients as compared to that in a risk-matched population. Higher leukocyte ROCK activity also predicts less reduction in 90-day NIHSS and is associated with lower stroke-free survival following an ischemic stroke event, while higher plasma hsCRP level predicts more severe neurological dysfunction at baseline and recurrent stroke. Therefore, both leukocyte ROCK activity and plasma hsCRP level can be used to predict outcomes following acute ischemic stroke.

## Supplementary Material

Supplemental Figure 1. Receiver-operating characteristic plot for the determination of cut-off values for rho kinase activity and high-sensitive CRP
(A) Receiver-operating characteristic plot for the cut-off value of hsCRP. The area under curve of plasma hsCRP level is 0.691 (p= 0.060).
(B) Receiver-operating characteristic plot for the cut-off value of ROCK activity. The area under curve of leukocyte ROCK activity is 0.704 (p= 0.021).

## Figures and Tables

**Table 1:** Demographics of risk-matched group and ischemic stroke group.

	Risk-matched Control group (*n* = 33)	Ischemic stroke group (*n* = 33)	*P* value
Age (years)	59.2 ± 8.3	57.5 ± 10.1	0.475
Male (%)	20 (60.6%)	22 (66.7%)	0.609
Body mass index	25.7 ± 3.4	24.9 ± 3.7	0.388
Hypertension (%)	18 (54.5%)	18 (54.6%)	1.000
Dyslipidemia* (%)	14 (42.4%)	12 (36.4%)	0.614
Diabetes mellitus (%)	9 (27.3%)	9 (21.2%)	0.566
Current smoker (%)	4 (12.1%)	18 (54.6%)	0.492
Number of risk factors	2.4 ± 1.1	2.4 ± 0.9	1.000

*Dyslipidemia: LDL > 160 mg/dL, triglyceride > 150 mg/dL, HDL < 40 mg/dL in male, or HDL < 50 mg/dL in female.

**Table 2:** Demographics of entire cohort in ischemic stroke group.

	Ischemic stroke patients (*n* = 110)
Age (years)	64.9 ± 12.1
Male (%)	77 (70.0%)
Risk factors	
Hypertension (%)	78 (70.9%)
Dyslipidemia* (%)	44 (40.0%)
Diabetes mellitus (%)	36 (32.7%)
Current smoker (%)	34 (30.9%)
History of atrial fibrillation	8 (7.3%)
TOAST etiology	
Macroangiopathy	40 (36.4%)
Microangiopathy	49 (44.5%)
Embolic	8 (7.3%)
Cryptogenic	13 (11.8%)
Hemorrhagic transformation	12 (10.9%)
Territory	
ACA	5 (4.5%)
MCA	65 (59.1%)
PCA and VB	28 (25.5%)
Undetermined	12 (10.9%)
NIHSS score at admission	6.4 ± 6.8
Medication at discharge	
Aspirin	80 (72.7%)
Clopidogrel	18 (16.4%)
Warfarin	2 (1.8%)
Beta blocker	17 (15.5%)
CCB	33 (30.0%)
ACEI/ARB	41 (37.2%)
Diuretics	2 (1.8%)
Statin	42 (38.2%)

*Dyslipidemia: LDL > 160 mg/dL, triglyceride > 150 mg/dL, HDL < 40 mg/dL in male, or HDL < 50 mg/dL in female.

ACA: anterior cerebral artery; ACEI: angiotensin converting enzyme inhibitor; ARB: angiotensin receptor blocker; CCB: calcium channel blocker; MCA: middle cerebral artery; NIHSS: National Institute of Health Stroke Scale; PCA: posterior cerebral artery; TOAST: acute stroke treatment; VBA: vertebrobasilar artery.

**Table 3:** Correlation between leukocyte ROCK activity, white blood cell count, and plasma levels of cytokines.

	TNF*α*	IL-6	hsCRP	WBC count
ROCK	0.017 (*P* = 0.870)	0.046 (*P* = 0.651)	−0.020 (*P* = 0.848)	0.029 (*P* = 0.764)
TNF*α*	1	0.242 (*P* = 0.016)	0.109 (*P* = 0.286)	−0.055 (*P* = 0.593)
IL-6	0.242 (*P* = 0.016)	1	0.565 (*P* < 0.001)	0.150 (*P* = 0.144)
hsCRP	0.109 (*P* = 0.286)	0.565 (*P* < 0.001)	1	0.235 (*P* = 0.022)

The numbers in each column denote the correlation efficient calculated by Spearman correlation test and the *P* value.

hsCRP: high sensitive C-reactive protein; IL-6: interleukin-6; ROCK: Rho kinase; TNF*α*: tumor necrotic factor alpha; WBC: white blood cell.

**Table 4:** A multivariate analysis by Cox proportional hazard model for predictors of recurrent stroke during follow-up.

Parameters	Hazard Ratio (95% CI)	*P* value
Entire ischemic stroke patient cohort (*n* = 110)		
hsCRP	1.214 (1.068–1.381)	0.003
Leukocyte ROCK activity	1.828 (0.536–6.289)	0.338
Atherosclerotic stroke patient cohort (*n* = 89)		
hsCRP	1.351 (1.056–1.728)	0.017
Leukocyte ROCK activity	5.207 (1.200–22.584)	0.028

hsCRP: high sensitive C-reactive protein; ROCK: Rho kinase.
