# Supplementary material for: The Prognostic Values of Leukocyte Rho Kinase Activity in Acute Ischemic Stroke
Source: Biomed Res Int. 2014 Feb 27;2014:214587. doi: 10.1155/2014/214587 (PMC3955656; doi:10.1155/2014/214587)
Supplement: Supplementary file 1 — Supplemental Figure 1. Receiver-operating characteristic plot for the determination of cut-off values for rho kinase activity and high-sensitive CRP (A) Receiver-operating characteristic plot for the cut-off value of hsCRP. The area under curve of plasma hsCRP level is 0.691 (p= 0.060). (B) Receiver-operating characteristic plot for the cut-off value of ROCK activity. The area under curve of leukocyte ROCK activity is 0.704 (p= 0.021). [file 214587.f1.docx]

**Supplemental Figure 1. Receiver-operating characteristic plot for the determination of cut-off values for rho kinase activity and high-sensitive CRP**

(A)


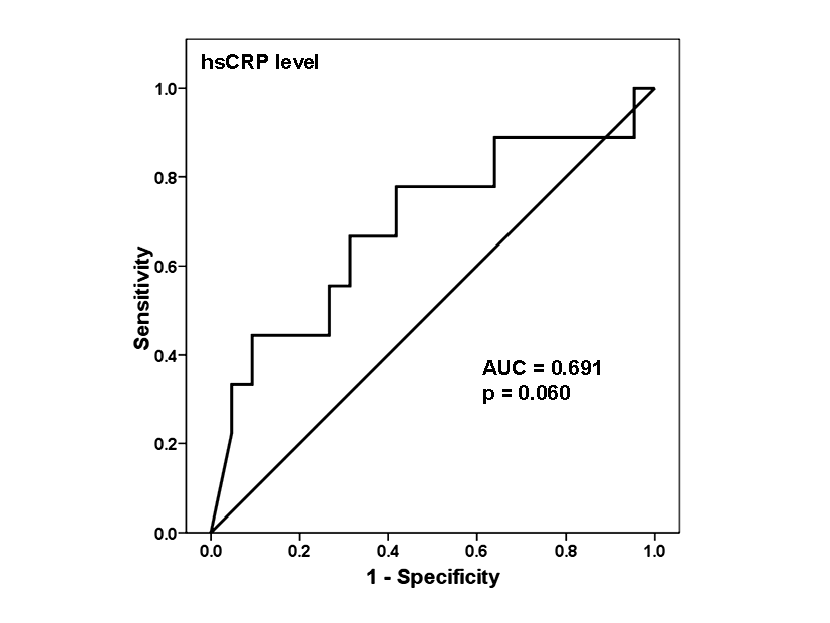


(B)


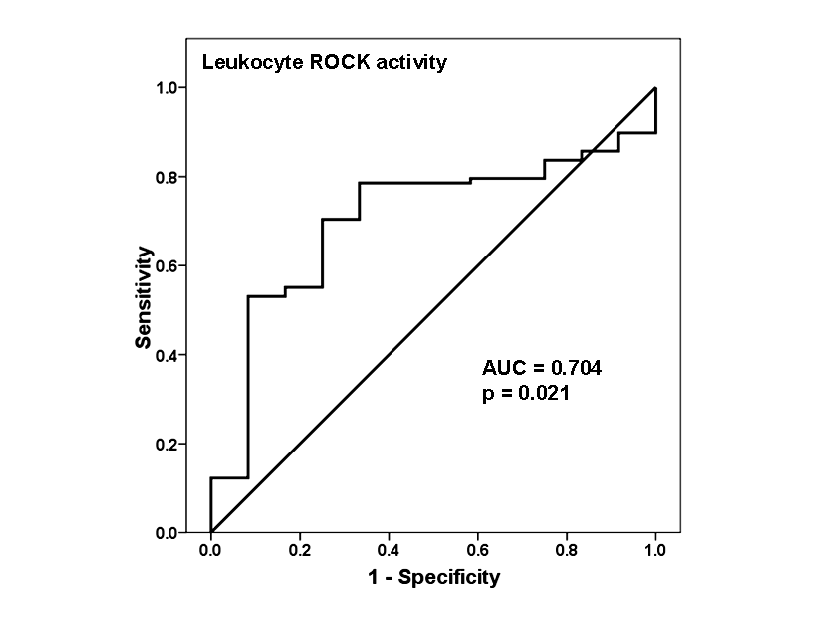


1. Receiver-operating characteristic plot for the cut-off value of hsCRP. The area under curve of plasma hsCRP level is 0.691 (p=0.060).
2. Receiver-operating characteristic plot for the cut-off value of ROCK activity. The area under curve of leukocyte ROCK activity is 0.704 (p=0.021).
